# Supplementary figures and images for: Inhibition of TXNRD or SOD1 overcomes NRF2-mediated resistance to β-lapachone
Source: Redox Biol. 2020 Jan 23;30:101440. doi: 10.1016/j.redox.2020.101440 (PMC6997906; doi:10.1016/j.redox.2020.101440)

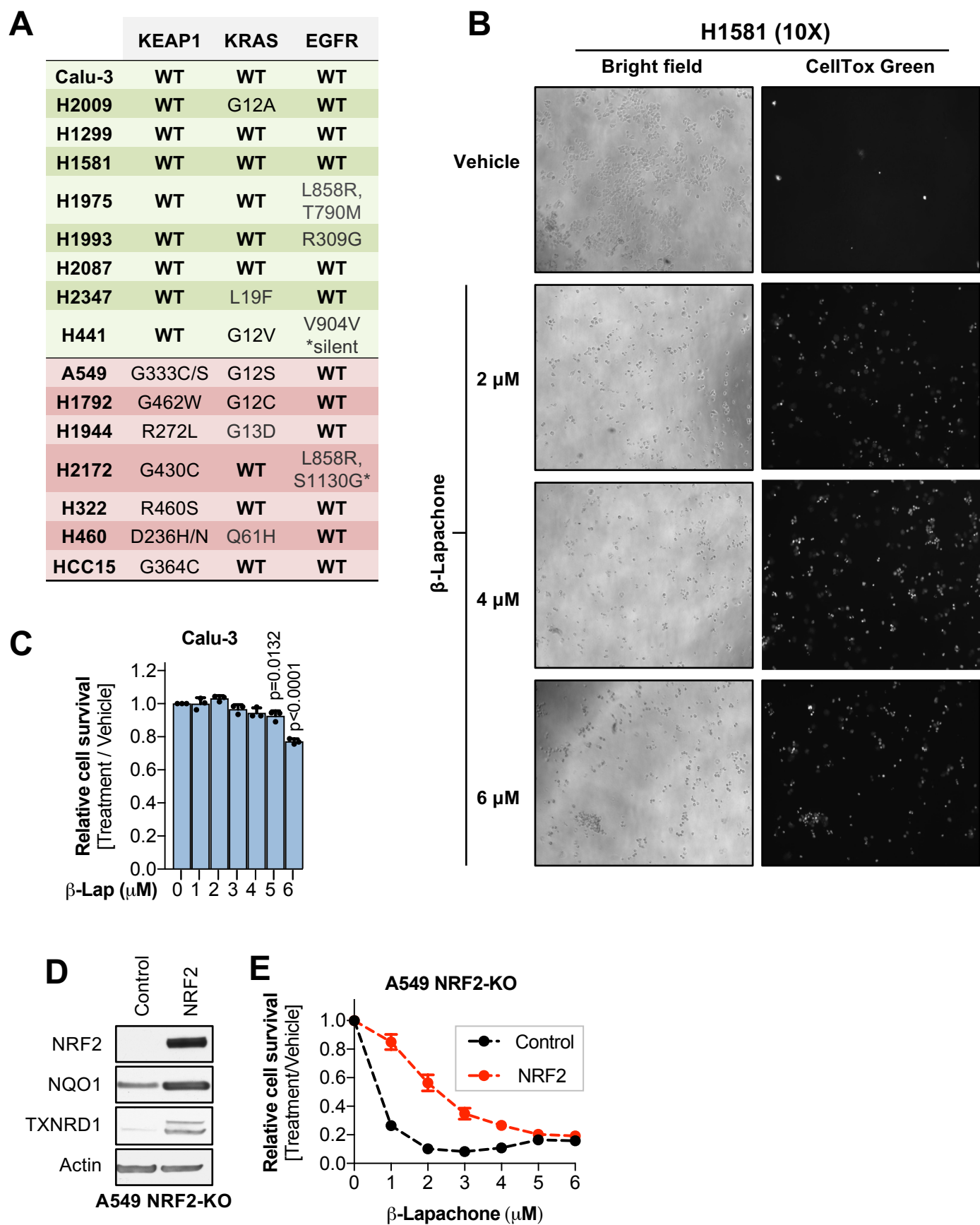

**Figure S1**

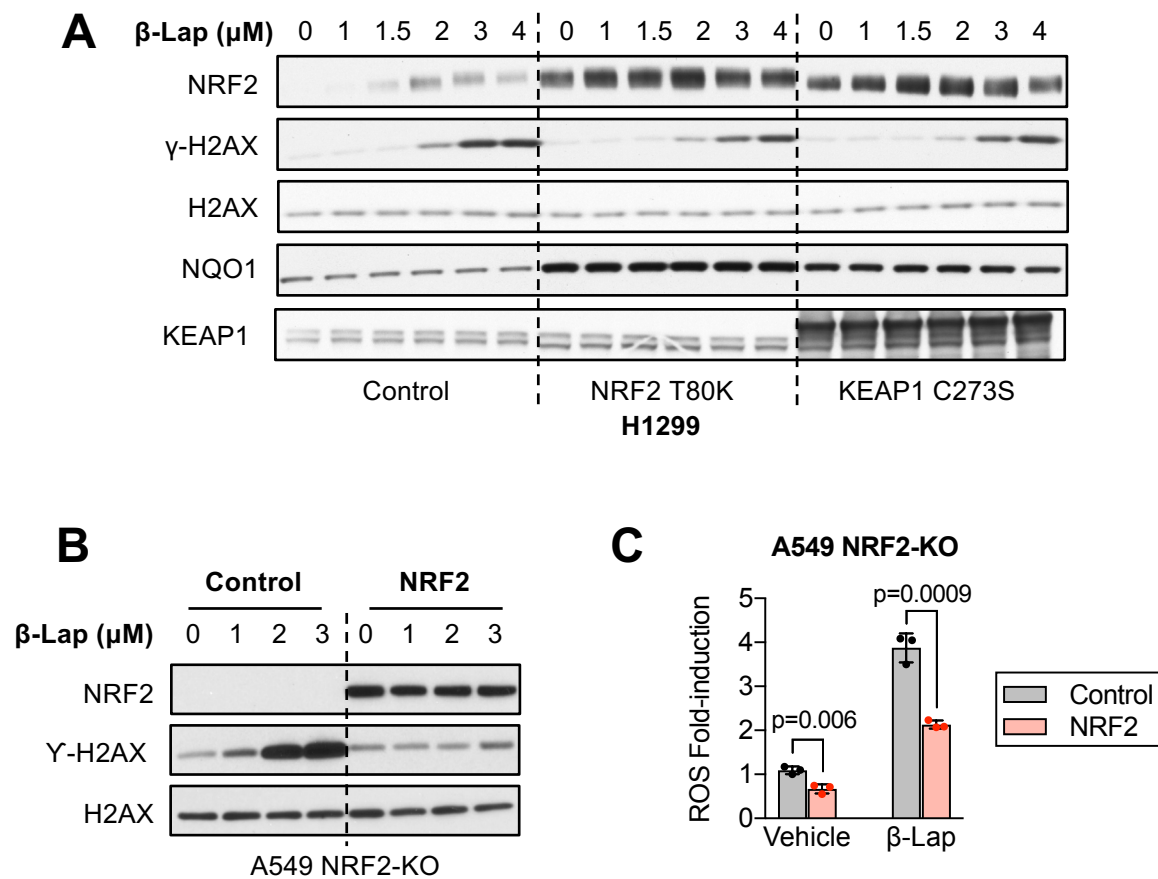

Figure S2

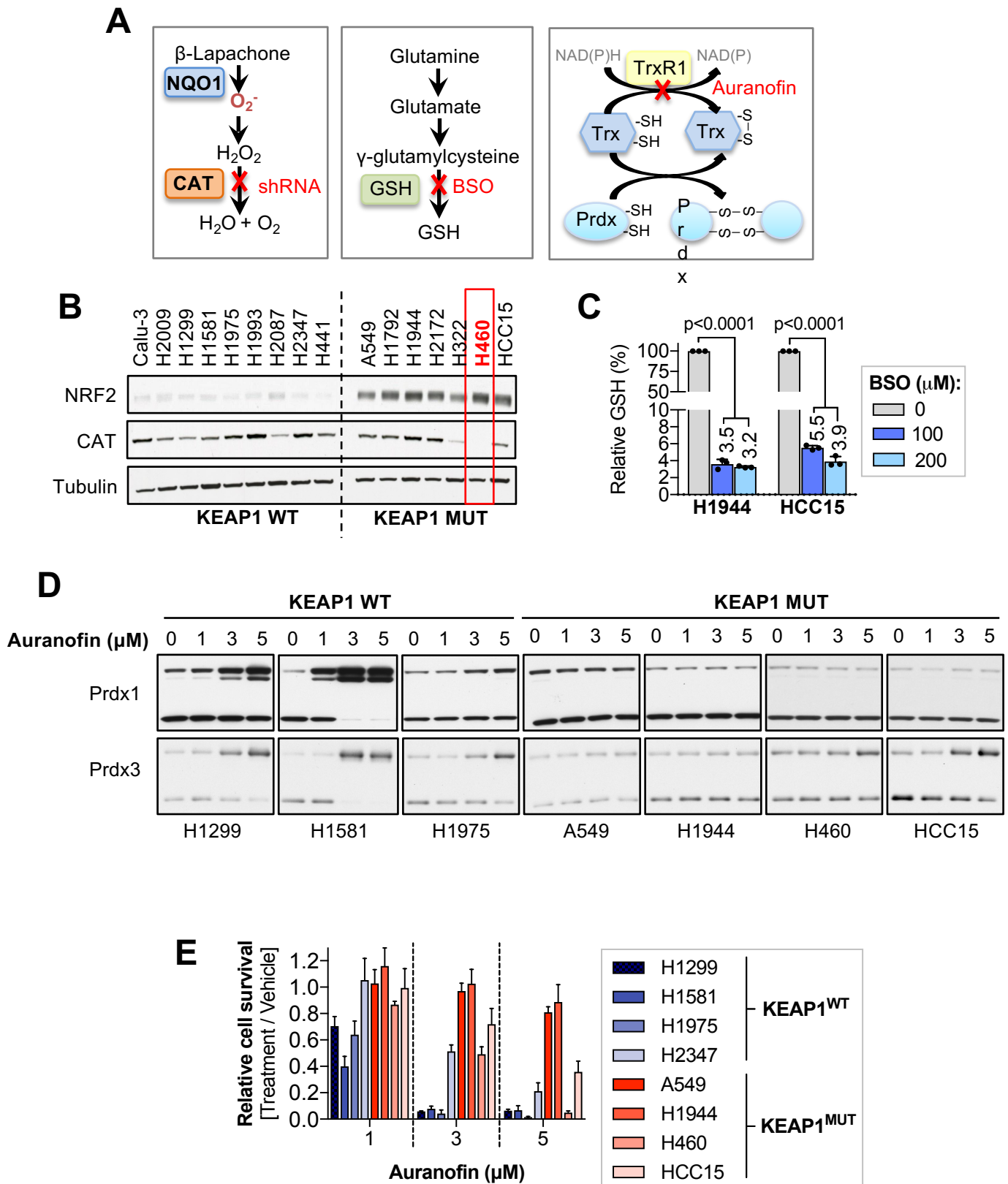

**Figure S3**

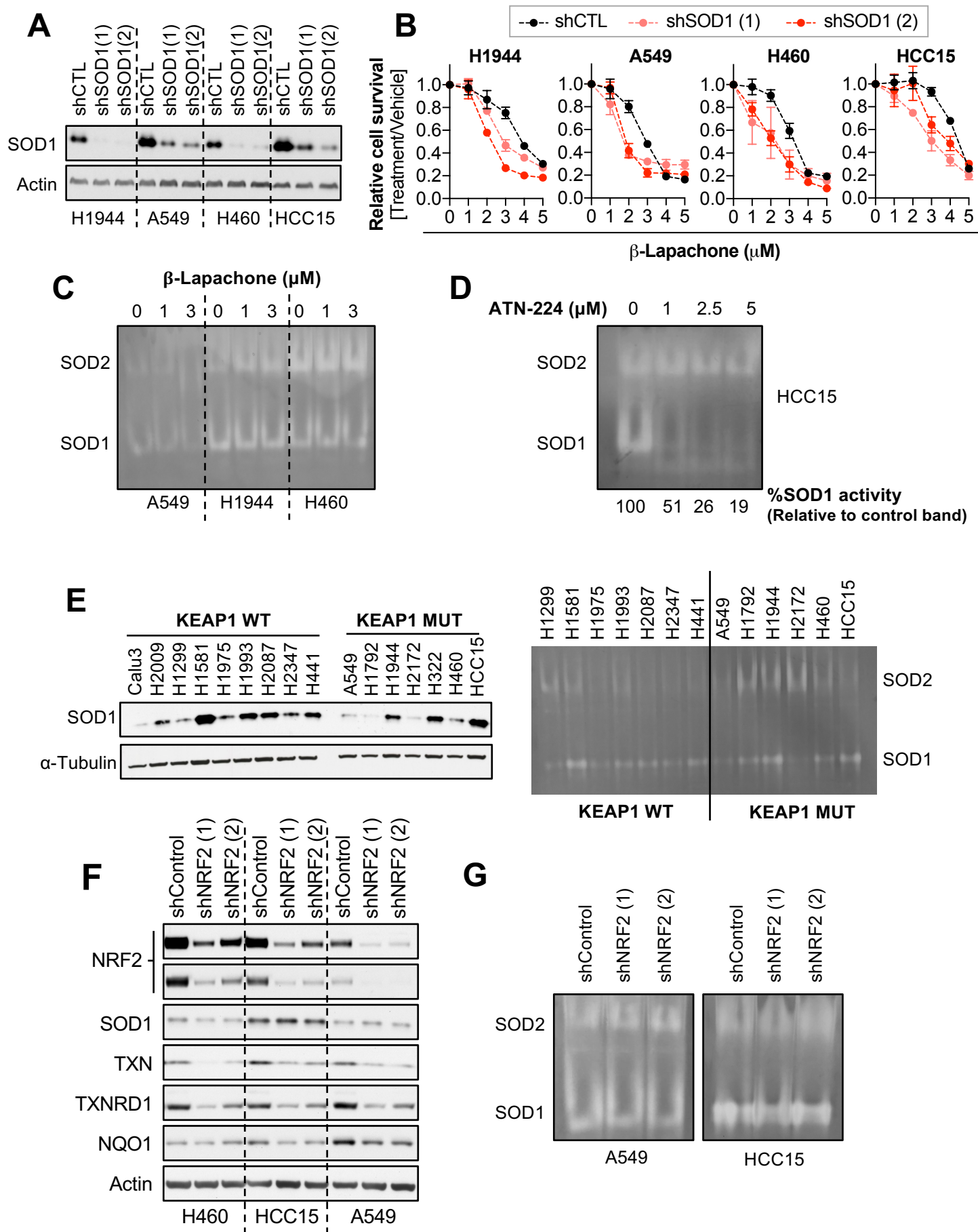

**Figure S4**

Supplement: Multimedia component 1 [file mmc1.pdf]
